# Raynaud's phenomenon in pediatric age

**DOI:** 10.1186/1546-0096-9-S1-P234

**Published:** 2011-09-14

**Authors:** M Tavares, A Novo, H Sousa, I Silva, I Almeida, M Guedes

**Affiliations:** 1 Serviço de Pediatria; 2 Serviço de Cirurgia Vascular; 3 Serviço de Medicina Interna, Centro Hospitalar do Porto

## Background

Raynaud's phenomenon (RP) is characterized by changes in the color of extremities: pallor, cyanosis and erythema. ItÂ´s prevalence in adults ranges between 3-20% but there are few studies in pediatric age. RP in boys or in children less than 12 years seems more associated to the secondary form.

## Aim

Assessment of RP in children and adolescents referred to Centro Hospitalar Porto, in the last 11 years.

## Methods

Retrospective analysis of clinical files of children and adolescents in which RP was the reason for consultation. Statistic analyses: SPSS version 19.

## Results

Sixty six cases (71,2% female) met the inclusion criteria. Average age of RP onset was 12,5y with median duration of 3,6y. RP was associated to: erythema pernio (34,8%), inter-phalangeal joints pain (30.3%), hands edema (28,8%), acrocyanosis (18,2%), profuse sweating (12,1% ) and trophic changes of fingers pulp (10,6%.). Antinuclear antibodies (ANA) were titled in 64 patients (97%), being positive (>1/80) in ten (15.6%). Capillaroscopy was performed on 43 (65.2%): 32 (74,4%) minor changes (tortuosity and segmental enlargement), four (9,3%) significant changes (giant capillaries, hemorrhagic and avascular areas), seven (16,3%) normal.

Of 43 patients with complete study, five (11,6%) had secondary RP: two with juvenile systemic sclerosis, two in characterization and a probable case of mixed connective tissue disease.

Secondary RP occurred mostly in less that 12-years-old child. ANA ≥ 1/320 and significant changes on capillaroscopy had a significant statistic relationship with secondary RP.

## Conclusion

RP can occur in children and, as in adults, in most cases is primary. ANAÂ´s positivity and changes in capillary bed were predictors of secondary RP.

